# Advance Care Planning and Frailty in Nursing Homes: Feasibility and Acceptance of a Stepwise, Long-Term Care, Structured Model

**DOI:** 10.3390/jcm15010214

**Published:** 2025-12-27

**Authors:** Miguel Sánchez Ortiz, Mercedes Forcano Garcia, Rogelio Altisent Trota, Javier Rocafort Gil

**Affiliations:** 1 Chair of Professionalism and Clinical Ethics, Aragon Health Research Institute (FEPS H36_23D), University of Zaragoza, 50009 Zaragoza, Spain; 2 Geriatrics Department, General University Hospital of Ciudad Real; 13004 Ciudad Real, Spain; 3 Geriatrics Department, Hospital Nuestra Señora de Gracia, 50004 Zaragoza, Spain; 4 Palliative Care Unit, Hospital San Juan de Dios, 31006 Pamplona, Spain; 5 Faculty of Health Sciencies, Francisco de Vitoria University, 28223 Madrid, Spain

**Keywords:** advance care planning, nursing homes, older adults, frailty, prospective study

## Abstract

**Background/Objectives**: Population aging in Europe presents significant healthcare, economic, and social challenges, particularly in the care of individuals with chronic diseases and frailty. Advance Care Planning (ACP) fosters patient autonomy and aligns end-of-life care with individual preferences. This study aimed to evaluate the acceptability and feasibility of an ACP model in nursing homes. Secondary objectives included exploring clinical characteristics of participants and assessing how frailty is associated with residents’ care goals and preferences. **Methods**: A prospective observational study was conducted among long-term residents of a Spanish nursing home in 2023. ACP was offered to all new permanent residents, with outcomes assessed through quarterly follow-ups. Acceptance rates, care preferences, and resident satisfaction were primary measures. Clinical data, frailty, functional status, cognitive assessments, and nutritional status were analyzed. The model of ACP is structured into three progressive levels: (1) identification of patients’ values, preferences, and global goals of care; (2) decision-making regarding specific clinical interventions in acute situations; and (3) end-of-life care preferences, including preferred place of death, desired companionship, and comfort-focused measures. **Results**: From 79 new residents admitted, 93.7% accepted ACP. The process required an average of 139 min to complete, distributed over 3–4 sessions. The main documented preferences included do-not-resuscitate orders (CPR) (79%), hospital transfer decisions (50%), and other individualized care choices. When stratified by frailty level, which was categorized as low, moderate, or high—we observed a clear gradient in care preferences. CPR preference increased from 59.3% (Low) to 87.5% (Moderate) and 95.2% (High). Preference to avoid hospital transfer rose from 22.2% to 50.0% and 85.7%, respectively. Avoidance of instrumentalization increased from 56.2% to 85.0% and 95.0%. **Conclusions**: ACP in nursing homes is highly acceptable and feasible, with benefits in aligning care with patient preferences and enhancing satisfaction.

## 1. Introduction

Population aging in Europe presents considerable healthcare, economic, and social challenges, especially in the care of individuals with advanced chronic diseases [1]. The World Health Organization (WHO) estimates by 2050, that over 30% of the European population will be over 65 years old, increasing the prevalence of chronic conditions and the need for complex clinical decision-making in advanced stages of life [2]. Connors et al. suggests that this group of older adults frequently faces unwanted hospitalizations and invasive procedures that may negatively impact their quality of life [3].

Advance Care Planning (ACP) has emerged as a structured process to foster patient autonomy and respect for preferences in end-of-life clinical decisions [4]. Since the mid-twentieth century, legislation and bioethics have promoted the inclusion of terms such as “limitation of therapeutic effort” and “advance directives” in medical practice, establishing a framework that protects patient autonomy and facilitates informed decision-making [5]. ACP has evolved to encompass not only medical preferences but also psychosocial and spiritual aspects, as well as the identification of a representative to act on the patient’s behalf if they lose their decision-making capacity [6].

Recent studies have demonstrated that ACP effectively reduces unwanted clinical interventions, decreases hospitalizations, and improves patient and family satisfaction [7,8]. In a systematic review, Brinkman-Stoppelenburg et al. [9] documented a significant association between ACP and an increased use of palliative care, enhancing both end-of-life quality of life and communication between patients and medical staff.

However, while ACP has been implemented in hospitals and home care settings, its application in nursing homes remains limited [10]. Despite a growing number of older adults residing in these facilities, and the fact that up to 15% of deaths in Spain and other European countries occur in nursing homes, the ACP framework in these settings is scarce [11,12]. The literature suggests that this gap is due, in part, to a lack of protocols adapted to geriatric care, barriers in staff training, and challenges in conducting in-depth conversations with patients experiencing cognitive decline [13,14].

The primary aim of this study is to evaluate the acceptability of an Advance Care Planning model in nursing homes.

The study also aims to explore mid-term satisfaction with care among residents who participate in the ACP intervention, providing insight into perceived quality and continuity of care. Furthermore, it intends to describe the clinical characteristics of residents who accept the ACP model compared to those who decline.

Finally, the findings of this research seek to provide evidence on how to improve respect for autonomy and optimize the quality of care for chronic illnesses and frailty in the residential care context.

## 2. Materials and Methods

### 2.1. Study Design

This study used a prospective observational design to evaluate the implementation of an Advance Care Planning model in a cohort of people newly admitted to the Instituto Aragonés de Servicios Sociales (IASS) Javalambre, a nursing home in Teruel, Spain. The ACP model was offered to all new residents beginning a permanent stay in the nursing home in 2023. Outcomes were evaluated among those who accepted ACP and those who did not to assess the feasibility of the model and its impact on quality of care and resident satisfaction.

Prior to the study, a review of the ACP literature was conducted to improve our model; this review was published in the “Revista Española de Geriatría y Gerontología” [15]. An educational video by Monica Lalanda has also been designed for residents and relatives to present the importance and practical aspects of ACP (available online: https://youtu.be/wi7rFI0VGQ4?si=C_mwaE9lS3QnGBe2 (accessed on 18 October 2025).

### 2.2. Population

The study population comprised all new long-term residents admitted to IASS Javalambre, a public institution collaborating with San José Hospital’s Geriatrics Department for coordinated clinical monitoring and specialized geriatric consultation. Inclusion criteria required residents to be in permanent residence for at least three months, while exclusions included temporary residents and individuals with cognitive impairment when in-person guardianship was unavailable. No age limit was applied.

### 2.3. Advance Care Planning Model

For this study, an adapted version of the “Escalera de Planificación Anticipada de Cuidados (EPAL)” model was employed, specifically designed for implementation in residential care facilities (i.e., nursing homes). This model is structured into three levels of depth in advance care planning, enabling clinical and care decisions to be tailored to patients’ individual preferences based on their health status, cognitive abilities, and personal values [16].

Level 1: This initial level involves identifying the patient’s values, preferences, and general goals regarding their future care. Key aspects explored at this stage include dignity, pain and symptom control, and the desire for companionship.

Level 2: This level focuses on decision-making for specific interventions in the event of acute situations, including the acceptance or refusal of cardiopulmonary resuscitation (CPR), instrumentalization, or hospital transfers.

Level 3: The final level delves deeper into end-of-life care preferences, addressing the preferred place of death, desired companionship, and comfort measures.

This stepwise approach facilitates a flexible and patient-centered process, promoting shared decision-making that aligns with the individual’s values and preferences.

### 2.4. Recruitment and Sample Size

Residents were recruited through scheduled consultations, in which the principal investigator provided in-depth explanations of the ACP process. Those who declined ACP were invited to participate in the control group after giving informed consent. Recruitment and data collection were conducted between May and December 2023, followed by a six-month follow-up period. With an anticipated 80% ACP acceptance rate among new residents, a sample size of 40 was calculated based on a 95% confidence level, 5% precision, and a 10% estimated attrition.

### 2.5. Data Collection and Variables

Primary variables included acceptance of participation in ACP, care preferences, and resident satisfaction ratings. Clinical and demographic data were obtained from interviews and medical records, with specific assessments covering functional, cognitive, and nutritional status, as well as levels of social support. Frailty was measured with VGI-frail (0–1 scale); residents were grouped according to Low/Moderate/High levels of frailty [17]. Data was collected through quarterly follow-ups and updated with changes in clinical status, following a progressive approach to ACP decision-making. This progressive model progressed from initial discussions of ACP concepts to detailed planning phases, with periodic reviews to ensure alignment with residents’ changing needs.

### 2.6. Data Availability

All study data were stored in a secure, password-protected electronic database hosted on encrypted institutional servers belonging to the Hospital San José Teruel (Geriatrics Unit). Access was restricted to authorized members of the research team. Data were anonymized before analysis, and all personal identifiers were removed to ensure participant confidentiality. The dataset generated and analyzed during the current study is available from the corresponding author upon reasonable request. Data were managed using Microsoft Excel version 2511 (build 19426.20218; Microsoft Corp., Redmond, WA, USA). It has been submitted alongside the manuscript and will be accessible upon publication to ensure transparency and facilitate reproducibility of the findings.

### 2.7. Statistical Analysis

Descriptive analysis included frequency distribution for categorical variables and mean/median values for continuous variables, assessed for normality. Categorical comparisons employed chi-square or Fisher’s exact tests, while *t*-tests or ANOVA were used for continuous data, supplemented by Mann–Whitney or Kruskal–Wallis tests for non-normally distributed variables. Multivariate regression adjusted for potential confounders, with significance set at *p* < 0.05.

### 2.8. Ethical Considerations

The study complies with all Human Ethics and Consent to Participate regulations, in addition to the Helsinki Declaration. The study was approved by the Ethics and Research Committee of Aragon, Spain (CEICA; C.I. PI23/194, Act nº 08/2023). Funding statement in the manuscript: This research was funded by the Feminization of Health Professions Research Group (FEPS, H36_23D), which belongs to the Department of Employment, Science and Universities of the Government of Aragon, Spain.

## 3. Results

### 3.1. Participation in the Advance Care Planning Program

Of the 79 patients included, 74 (93.7%) accepted to participate in the Advance Care Planning program. The ACP model was successfully implemented in 90.5% of cases, where “completion” denotes the engagement in all three levels of our structured, stepwise care approach.

The average time required to complete the ACP process was 139 min (SD 42.5), with recorded durations ranging from 55 to 233 min. The mean time per session was 42 min (SD 9.9), and patients underwent an average of 3.4 sessions (SD 1.03) to finalize ACP.

### 3.2. Comprehensive Geriatric Assessment

Among the 79 patients included (Table 1), 61% were female, with a mean age of 87 years (SD 7). The youngest person was 65 years old and the oldest was 97 years old. In terms of clinical history, 86% had cardiovascular risk factors, 46% a history of cardiac conditions, 49% respiratory conditions, 78% neurological conditions, 58% digestive diseases, and 48% renal disease.

The Barthel Index [18] indicated an average functional dependency score of 38 (SD 31). Functional dependency levels were distributed as follows: 42% were fully dependent in basic activities of daily living (ADL), 25% had mild dependency, 19% moderate dependency, and 12% severe dependency. The mean gait speed was over 4 m 9.6 s (SD 4.7). A history of falls was reported by 39% of patients, with mobility aids used by 52% (cane or walker) and 20% (wheelchair).

Cognitive assessment, as measured by the Mini Mental State Examination (MMSE) [19], showed an average score of 17 (SD 7), with 13% of patients scoring within the normal range, 20% with mild cognitive impairment, 24% with moderate impairment, and 43% with severe cognitive impairment.

The mean score on the Mini Nutritional Assessment-Short Form (MNA-SF) [20] was 9.1 (SD 3.2), with 29% classified as nutritionally normal, 38% at risk of malnutrition, and 33% identified as malnourished. Dysphagia was present in 38% of patients. Other geriatric syndromes included constipation (41%), urinary incontinence (76%), and pressure ulcers (33%).

On the VGI-frail test, patients scored an average of 0.49 (SD 0.2), with patients categorized into three care goal groups: symptom management (47%), functional support (34%), and survival-oriented care (19%). Moreover, when categorized into tertiles, 33% of residents were classified as Low frailty (mean 0.35), 33% as Moderate frailty (mean 0.50), and 34% as High frailty (mean 0.66).

In terms of social support, the primary support was from children (76%), followed by spouses (5.1%) and other family members or acquaintances (3.8%). Notably, 15% lacked social support entirely.

### 3.3. Patient Preferences and Decisions

In 97.3% of cases, a designated guardian or responsible party was identified, of whom 63% were female. Health issues were documented for all patients (100%), and care preferences were recorded in 97% of patients. Core values expressed included dignity in care, companionship during illness, control over suffering and symptoms, spirituality, and decisions regarding end-of-life care and use of medical instrumentation.

**Figure 1:**
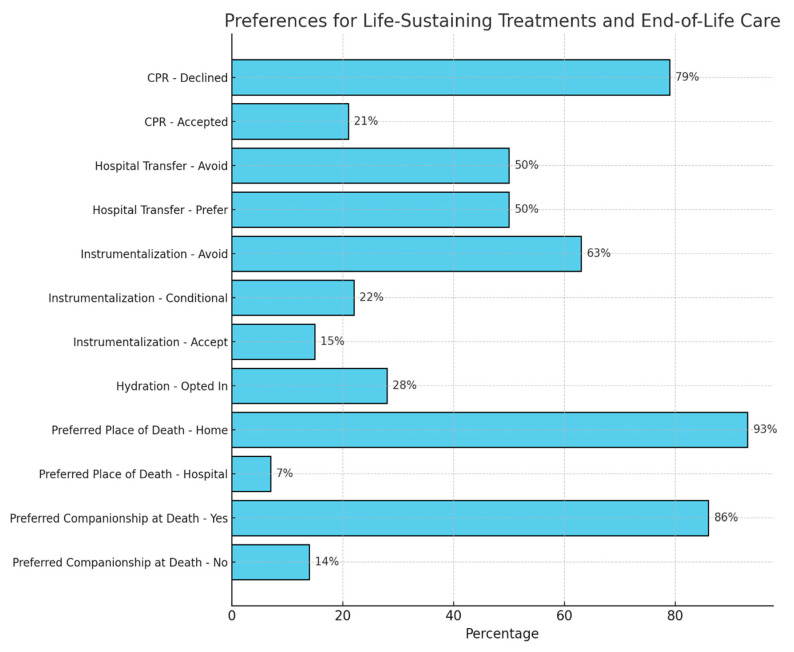
Care preferences and decisions.

Regarding specific clinical decisions (Figure 1), 79% of patients preferred to avoid cardiopulmonary reanimation (CPR), and 50% expressed a preference against hospital transfers. Additionally, 63% opted out of any form of life-sustaining instrumentation (e.g., intensive care, orotracheal intubation, mechanical ventilation, dialysis, non-ambulatory surgeries), while 22% preferred conditional acceptance based on specific acute conditions, and 15% agreed to full intervention if needed. Only one patient chose PEG feeding, while 28% accepted subcutaneous hydration.

The preference to die in their current residence was expressed by 93% of patients, while 86% wished to be accompanied by family or close friends at the time of death.

### 3.4. Analysis of Clinical Characteristics and Medical Care Preferences

With a *p*-value < 0.05, patients who declined CPR had a higher mean age (88 years, SD 7) compared to those opting for reanimation (83 years, SD 8). The probability of rejecting reanimation increased with age (OR 0.92; 95% CI: 0.85–0.98), while higher SPPB scores were associated with a greater likelihood of choosing CPR (OR 1.3; 95% CI: 1.09–15.7).

Barthel Index scores indicated that patients who preferred reanimation had a mean score of 62, whereas those who preferred to avoid it averaged 33 points. Lower Barthel scores (indicating greater dependency) were associated with a decreased desire for reanimation (OR 1.03; 95% CI: 1.01–1.05).

Regarding care goals, 53% of patients with survival-oriented goals opted for reanimation, compared to 40% with functional goals and only 6.7% with symptom-focused goals.

For hospital transfers, each additional year of age decreased the likelihood of choosing transfer (OR 0.86; 95% CI: 0.78–0.94). Higher SPPB scores were correlated with a greater likelihood of preferring hospital transfer (OR 1.4; 95% CI: 1.16–1.73). Greater dependency in ADLs was associated with a preference to avoid hospital transfers (OR 9.8; 95% CI: 2.5–38). A higher MMSE score, and absence of dysphagia or pressure ulcers, correlated with a greater desire for hospital transfer.

The likelihood of opting for instrumental care was higher in patients without respiratory history (OR 0.33; 95% CI: 0.12–0.89) and with higher Barthel Index scores (OR 1.02; 95% CI: 1.01–1.04). Among patients with major neurocognitive disorders, 78% preferred to avoid instrumentalization (OR 0.27; 95% CI: 0.09–0.73). Better nutritional status also increased the likelihood of accepting instrumentalization (OR 1.58; 95% CI: 1.28–2.07).

### 3.5. Analysis of Frailty and Medical Care Preferences

Frailty demonstrated a graded association with clinical preferences and care goals (Table 2). As frailty increased from Low to Moderate and High tertiles, the proportion of residents expressing Do-Not-Resuscitate preferences rose from 59.3% to 87.5% and 95.2%, respectively (OR 10.79; *p* = 0.0046). Similarly, the tendency to avoid hospital transfer increased from 22.2% to 50.0% and 85.7% (OR 19.05; *p* < 0.001), and avoidance of instrumental interventions rose from 56.2% to 85.0% and 95.0% (OR 8.88; *p* = 0.0118).

No significant associations were observed for parenteral hydration (OR 2.6; *p* = 0.27) or artificial nutrition (OR 1.1; *p* = 0.57).

Care goals also shifted markedly with frailty (OR 60.07; *p* < 0.001): symptom-focused goals predominated among highly frail residents, whereas functionality and survival goals were more frequent in Low and Moderate frailty categories.

In multivariate logistic regression models adjusted for age, sex, functional status (Barthel Index), cognitive performance (MMSE), and nutritional state (MNA-SF), VGI-frail remained an independent predictor of both decision preferences and care goals.

Each 0.1-point increase in the VGI-frail score was associated with 1.42-fold higher odds of preferring a CPR order (OR 1.42; 95% CI 1.08–1.92; *p* = 0.012). Higher frailty also predicted avoidance of hospital transfers (OR 1.56; 95% CI 1.22–2.13; *p* = 0.004) and instrumental interventions (OR 1.48; 95% CI 1.11–2.01; *p* = 0.017).

For care goals, each 0.1-point increase in VGI-frail was associated with a shift from functionality- to symptom-oriented goals (OR 2.11; 95% CI 1.45–3.67; *p* < 0.001).

These findings remained consistent across sensitivity analyses excluding residents with severe cognitive impairment or malnutrition.

### 3.6. Perceived Improvement and Satisfaction

Patient satisfaction with discussions on future care was rated on a visual numerical scale (1 to 10), with a mean score of 9.01 (SD 0.97) and a median of 9. In response to the question, “Do you believe our ACP model enhances care quality?” 100% of participants responded affirmatively.

Furthermore, 78.1% reported that discussions about future care did not induce distress. In terms of perceived improvement, patients reported gains in mobility (91%), self-care (63%), daily activities (64%), pain management (93%), and emotional well-being (97%).

## 4. Discussion

The implementation of the Advance Care Planning program in a geriatric population demonstrated high acceptance, with 93.67% of patients actively participating in planning their future care. This level of acceptance aligns with previous studies showing a growing interest and adherence to advance care planning programs in aging populations, where patients value the opportunity to express their wishes regarding end-of-life care [21,22]. The high completion rate of the ACP program (90.54%) suggests that this model, structured into three progressive stages, is effective in guiding patients through complex decision-making processes, as also reflected in similar studies in geriatric contexts [23,24].

The average time required to complete the ACP process (139 min, SD 42.5) and the mean of three interviews per patient indicate a deliberative and thorough approach that adequately addresses the patient’s needs and preferences, like the study by Gain-za-Miranda et al. [25]. The literature supports that dedicating adequate time to the ACP process contributes to higher quality decision-making, particularly in patients with significant comorbidities [26,27]. These findings suggest that ACP programs must allocate sufficient resources to ensure patients can reflect on and discuss their options in a safe and unhurried environment.

The initial geriatric assessment revealed that 86% of patients had cardiovascular risk factors, 78% had neurological conditions, and 48% had renal disease. These data reflect the common multimorbidity profile in geriatric patients and emphasize the need to incorporate ACP as part of standard care in geriatrics, where chronic conditions and functional dependency are prevalent [28,29]. The mean score of 38 on the Barthel Index, along with 42% of patients classified as totally dependent on basic activities of daily living, reinforces the necessity of such planning, as patients with greater functional dependency tend to prefer limiting aggressive interventions at the end of life [27,30].

Regarding cognitive assessment, 43% of patients were found to have severe cognitive impairment. This finding is consistent with studies showing a high prevalence of cognitive impairment in older adults and underscores the importance of initiating ACP early, before cognitive decline limits the patient’s capacity for autonomous decision-making [23,31]. In this study, the ACP process was conducted with the active involvement of the primary family caregiver, ensuring that the patient’s preferences and values were incorporated to the greatest extent possible. This approach aligns with best practices in shared decision-making and respects the patient’s autonomy despite their cognitive limitations. The loss of significance in multivariate analysis suggests that these decisions are influenced by a combination of factors beyond the primary disease state.

In terms of patient preferences, 79% rejected cardiopulmonary resuscitation (CPR), 50% preferred to avoid hospital transfers, and 63% declined instrumental interventions. These results align with previous studies showing a tendency among geriatric patients to prioritize quality of life and minimize invasive interventions, particularly in cases of multi-morbidity and frailty [21,27]. Moreover, the fact that 93% of patients expressed a desire to die in their residence, surrounded by loved ones (86%), aligns with the goals of palliative care to enhance quality of life and emotional well-being in advanced stages [24,28].

Overall satisfaction with the ACP program was high, with an average score of 9.01 out of 10. All participants stated that the ACP model improves the quality of care received, reinforcing the relevance of these programs in geriatric care. Additionally, 78.1% of patients indicated that discussing future care did not induce distress, which is consistent with studies suggesting that advance care planning, rather than increasing anxiety, provides patients with a sense of control and reassurance [23,27].

Finally, the main limitation of this study is that it was performed in a single nursing home in Spain, which may limit the generalizability of the results to other populations. Another limitation is the use of self-reported measures of satisfaction and perceived benefit, which introduce subjective variability and may be influenced by the emotional or cognitive state of the participants. The study did not evaluate longitudinal clinical or functional outcomes related to advanced care planning decisions, such as hospitalizations or treatment appropriateness, which warrants further investigation.

## 5. Conclusions

This study describes that implementing an Advance Care Planning model in nursing homes is both feasible and highly acceptable among geriatric residents.

The high advanced directive completion rate further supports the practicality and effectiveness of this structured approach in long-term care settings.

The findings suggest that older adults value the opportunity to actively engage in planning their care, aligning medical interventions with their personal preferences and values.

The structured, stepwise approach employed in this model facilitated a comprehensive exploration of patients’ medical, psychosocial, and spiritual needs, contributing to the high completion rate and overall satisfaction with the process.

The study also highlights key factors influencing care preferences, including age, functional dependency, cognitive status, and frailty.

Notably, higher frailty was associated with a greater preference for end-of-life comfort measures, underscoring the importance of integrating frailty assessment into ACP discussions.

The authors recommend, at an educational level, promoting the routine integration of Advance Care Planning (ACP) in nursing homes to ensure the systematic documentation of residents’ values and care preferences.

## Figures and Tables

**Table 1:** Demographic and clinical characteristics.

Characteristic	Value (%)	Mean (SD)
Number of Patients	79	
Female Patients	61%	
Mean Age (SD)		87 (7)
Cardiovascular Risk Factors	86%	
Cardiological History	46%	
Pulmonary History	49%	
Neurological History	78%	
Digestive Diseases	58%	
Renal Disease	48%	
Barthel Index Score		38 (31)
Dependency Levels		
Total Dependency	42%	
Severe Dependency	12%	
Moderate Dependency	19%	
Mild Dependency	25%	
MMSE Score		17 (7)
Cognitive Status		
Normal	13%	
Mild Cognitive Impairment	20%	
Moderate Cognitive Impairment	24%	
Severe Cognitive Impairment	43%	
MNA-SF Score		9.08 (3.16)
Nutritional Status		
Normal Nutrition	29%	
Risk of Malnutrition	38%	
Malnutrition	33%	
Dysphagia	38%	
Constipation	41%	
Urinary Incontinence	76%	
Pressure Ulcers	33%	

**Table 2:** Association between frailty and clinical preferences.

Preference	Low Frailty	Moderate Frailty	High Frailty	OR	*p*
**Do-Not-Resuscitate**	59.3%	87.5%	95.2%	10.79	0.0046
**Avoid hospital Transfer**	22.2%	50.0%	85.7%	19.05	<0.001
**Avoid instrumental interventions**	56.2%	85.0%	95.0%	8.88	0.0118

## Data Availability

The original contributions presented in this study are included in the article. Further inquiries can be directed to the corresponding author.

## Abbreviations

The following abbreviations are used in this manuscript:

ACP	Advance Care Planning
CPR	Cardiopulmonary Resuscitation
WHO	World Health Organization
ADL	Activities of Daily Living
MMSE	Mini-Mental State Examination
MNA-SF	Mini Nutritional Assessment—Short Form
SPPB	Short Physical Performance Battery
EPAL	Ladder for Advance Care Planning
VGI-frail	Comprehensive Geriatric Assessment—Frailty Index
ABVD	Basic Activities of Daily Living
CEICA	Aragon Research Ethics Committee
OR	Odds Ratio
CI	Confidence Interval
SD	Standard Deviation
ANOVA	Analysis of Variance
IASS	Aragon Institute of Social Services
HCP	Health Care Professionals
